# A pediatric case of primary amoebic meningoencephalitis due to *Naegleria fowleri* diagnosed by next-generation sequencing of cerebrospinal fluid and blood samples

**DOI:** 10.1186/s12879-021-06932-9

**Published:** 2021-12-14

**Authors:** Shiqin Huang, Xiu’an Liang, Yunli Han, Yanyan Zhang, Xinhui Li, Zhiyong Yang

**Affiliations:** 1 Department of Pediatrics, The First Affiliated Hospital of Guangxi Medical University, 6 Shuangyong Road, Qingxiu District, Nanning, China; 2 Guangzhou Sagene Biotech Co., Ltd., Guangzhou, China

**Keywords:** *Naegleria fowleri*, Cerebrospinal fluid, Blood, Primary amoebic meningoencephalitis, Meta-genomics next generation sequencing

## Abstract

**Background:**

Primary amoebic meningoencephalitis (PAM) is a rare, acute and fatal disease of the central nervous system caused by infection with *Naegleria fowleri* (Heggie, in Travel Med Infect Dis 8:201–6, 2010). Presently, the majority of reported cases in the literature have been diagnosed through pathogen detection pathogens in the cerebrospinal fluid (CSF). This report highlights the first case of pediatric PAM diagnosed with amoeba infiltration within CSF and bloodstream of an 8-year-old male child, validated through meta-genomic next-generation sequencing (mNGS).

**Case presentation:**

An 8-year-old male child was admitted to hospital following 24 h of fever, headache and vomiting and rapidly entered into a coma. CSF examination was consistent with typical bacterial meningitis. However, since targeted treatment for this condition proved to be futile, the patient rapidly progressed to brain death. Finally, the patient was referred to our hospital where he was confirmed with brain death. CSF and blood samples were consequently analyzed through mNGS. *N. fowleri* was detected in both samples, although the sequence copy number in the blood was lower than for CSF. The pathogen diagnosis was further verified by PCR and Sanger sequencing.

**Conclusions:**

This is the first reported case of pediatric PAM found in mainland China. The results indicate that *N. fowleri* may spread outside the central nervous system through a damaged blood–brain barrier.

## Background

Primary amoebic meningoencephalitis (PAM) is a fulminant, hemorrhagic and necrotizing meningitis [1], caused by *Naegleria fowleri* infection. This infection is very rare in China. The exact pathogenesis of PAM is unclear, though *N. fowleri* is often called a “brain-eating amoeba” since it causes severe encephalitis following infection, with a fatality rate of over 95% [2]. PAM is an acute and progressive process, with an incubation period ranging from 2 to 15 days. Patients usually die 3–7 days after the onset of symptoms [3].

The clinical manifestations of PAM are very similar to bacterial meningitis, which makes early diagnosis difficult. This report highlights the first case of pediatric PAM in China with positive cerebrospinal fluid (CSF) and blood that was consequently confirmed through meta-genomic next-generation sequencing (mNGS).

## Case presentation

Ethical approval for this study was obtained from the Research Ethics Committee of the First Affiliated Hospital of Guangxi Medical University. The patient was an 8-year-old male admitted to hospital due to headaches, vomiting and fever during the previous 24 h. In addition, the patient presented disturbance of consciousness for 22 h prior to admission. Following symptomatic treatment, vomiting was relieved although the patient still complained of fever, with the highest body temperature recorded at 39.8 ℃, together with persistent and severe headaches. The patient was hospitalized and consequently developed status epilepticus within 24 h of being admitted to hospital. The patient consequently became unconscious and required treatment with a tracheal intubation ventilator.

**Fig. 1:**
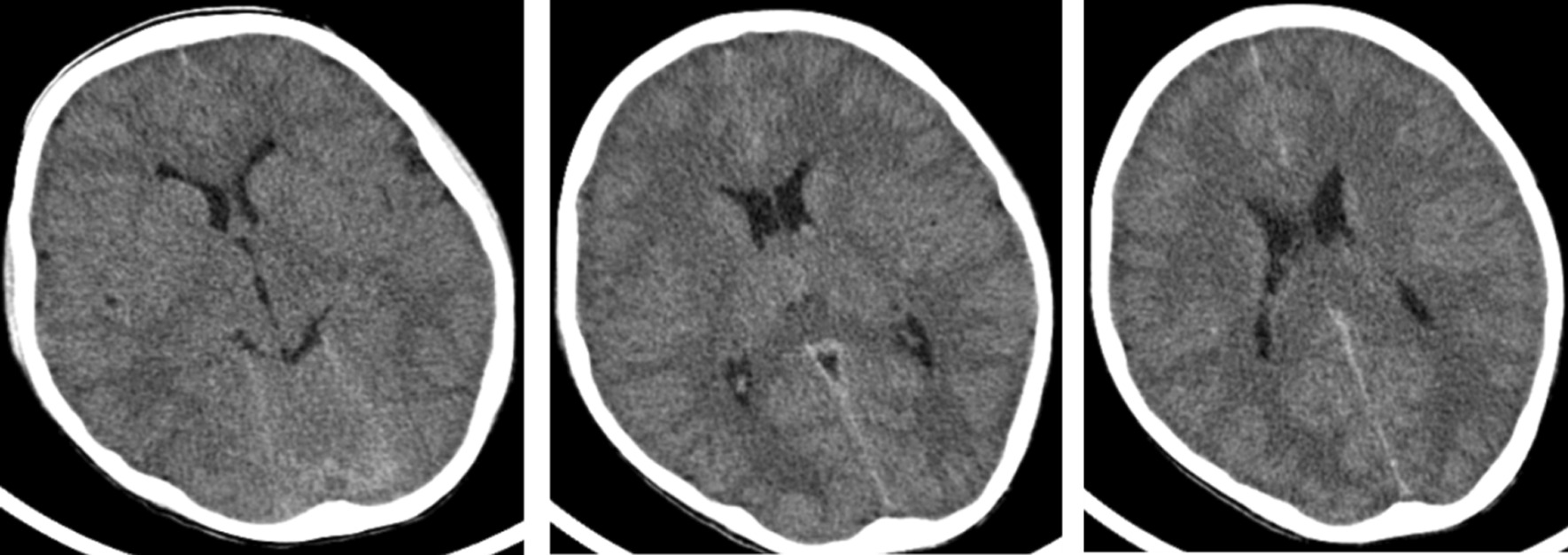
Patient cranium CT scans. Local density of cerebral sickle and tentorium slightly increased, subarachnoid hemorrhage was not excluded

The clinical blood analyses performed consisted of the following: WBC 19.39 * 10^9^/L, N% 90.2%, Hb 120 g/L, PLT 241 * 10^9^/L, CRP 57.6 mg/L. Lumbar puncture was performed, and the test results for CSF are listed in Table 1. Head CT scans identified a local density of cerebral sickle and the tentorium was slightly increased. Subarachnoid hemorrhage was not excluded (Fig. 1). The patient was given meropenem/vancomycin as an antibiotic treatment, methylprednisolone sodium succinate as an anti-inflammatory treatment, midazolam/propofol as sedative and anti-convulsion treatment, sodium valproate to control epilepsy together with active treatment to lower intracranial pressure.

**Table 1:** Clinical test results for the cerebrospinal fluid sampled on two occasions

	June 17, 2020	June 23, 2020	Reference values
ICP (mmH_2_O)	270	300	70–180
WBC (× 10^6^/L)	212	4134	0–5
Percentage of neutrophils	51%	90%	0–2%
Monocytes	49%	10%	Variable
RBC (× 10^6^/L)	15%	35%	0
Glucose (mmol/L)	0.5	0.22	2.2–4.4
Glucose ratio in the cerebrospinal fluid to the blood	0.08	0.04	> 0.6
Protein (mg/L)	2826	18,740	< 0.4

All values were measured in duplicates. ICP: intracranial pressure; WBC: Leucocyte; RBC: red blood cells (both cerebrospinal fluid examinations showed marked leukocytosis and erythrocytosis, hypoglycemia and elevated protein levels together with neutrophilic predominance of pleocytosis)

Seven days later, spontaneous breathing halted and the patient fell into a deep coma with a Glasgow score of 3.0. Consequently, he was referred to the Provincial Maternal and Child Health Hospital for hospitalization. An additional lumbar puncture was performed while CSF was re-tested (Table 1). A CSF smear/culture were performed in conjunction with gamma interferon and seven viral nucleic acid tests—no abnormalities were denoted.

**Fig. 2:**
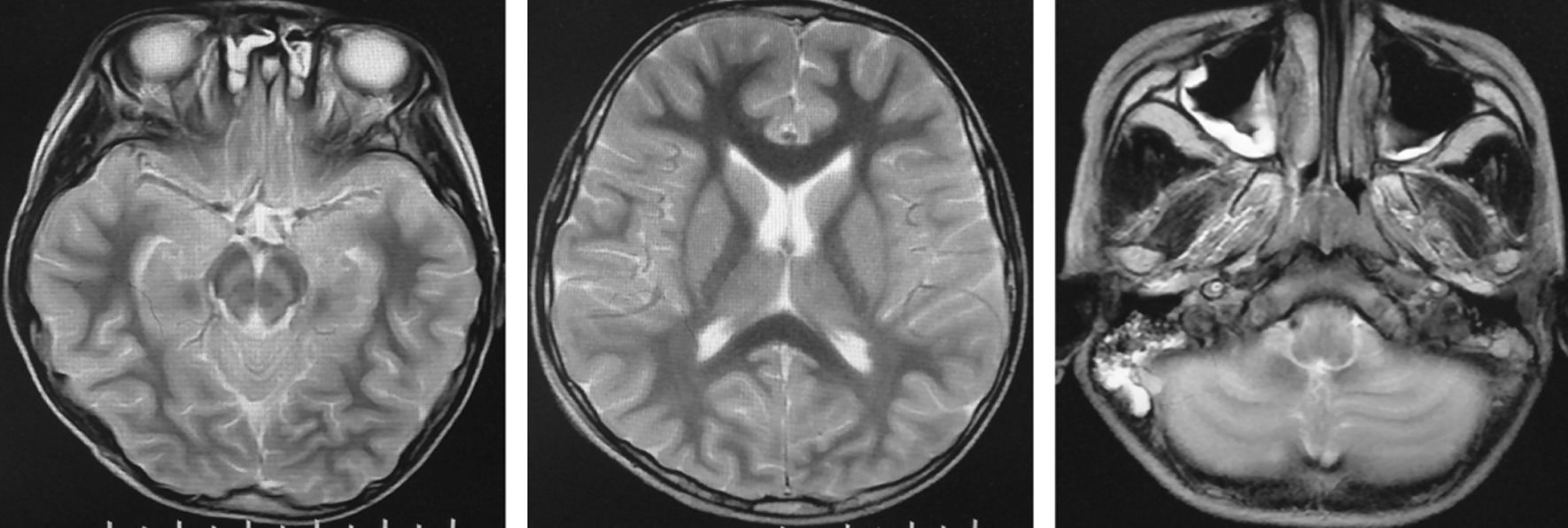
Patient head/neck MRI scans of the patient. Images exhibit diffuse swelling with abnormal signals in the bilateral cerebral hemispheres, basal ganglia, thalamus, brainstem, cerebellar hemispheres and also highlights extensive cerebral edema. The fourth ventricle, ring cistern, pontine cistern and extra-cerebral space are narrow and the lower edge of the cerebellar tonsils have become pointed and moved down slightly

Head and neck magnetic resonance imaging (MRI) was performed on the 10th day post-admission. The bilateral cerebral hemispheres, basal ganglia, thalamus, brainstem and cerebellar hemispheres were found to be diffusely swollen with abnormal signals, and extensive cerebral edema was considered. Observed abnormal narrowing of the fourth ventricle, cisterna, pontine cistern, and extracerebral space, and the lower edge of the cerebellar tonsils (Fig. 2).

The patient was still treated with a broad-spectrum anti-infective treatment, based on a diagnosis of bacterial meningitis. This treatment included meropenem, vancomycin and ceftriaxone, although all proved to be ineffective.

The patient was subsequently transferred to the First Affiliated Hospital of Guangxi Medical University, 24 days after onset of the condition and was deemed to be ‘brain dead’ according to the Chinese Children’s Brain Death Judgment Standard [4]. A lumbar puncture was repeated, with a challenging puncture, and with only a minute amount of pale, pink, thick necrotic fluid drawn out from the syringe. The CSF, together with a blood sample were sent for mNGS [RDP-seq^®^, Guangzhou Sagene Biotechnology Co., Ltd.]. The plasma was firstly separated from blood at 3000 rpm for10 min and consequently centrifugated at 15,000 rpm for 5 min. This was followed by nucleic acid extraction and purification with a nucleic acid extraction kit combined with magnetic beads [Sagene™, Guangzhou, CHINA]. A 2 mL CSF sample was directly centrifugated at 15,000 rpm for 5 min, and then were used for nucleic acid extraction and purification with nucleic acid extraction kit, as for plasma sample. The library was constructed according to the protocol for library construction Kit [Nextera XT^®^, Illumina™, USA]. Once the libraries were mixed with equivalent amounts, high-throughput sequencing was performed on the Illumina™ Nextseq 550 DX^®^, sequencing platform (sequencing strategy: SE75), which is an FDA-approved and CE-IVD-certified sequencer. The results confirmed infection with *N. fowleri*.

A total 326 detected sequences were mapped to the *N. fowleri* genome (ASM1484362v1, genome size: 27.8 Mb) in CSF samples (total number of reads detected: 13,041,601). A total of 64 reads for the same pathogen were detected in blood samples (total number of reads detected: 15,659,034), and the analysis reliability was over 99%. Concomitantly, we analyzed the distribution map of the test sequences in each sample on the genome. The read coverage against the reference genome was 0.09% (CSF) and 0.02% (blood), respectively, with no overlap between the aligned sequence positions on the genome. PCR and Sanger sequencing were consequently employed [S-Reagent^®^ verification system, Guangzhou Sagene Biotechnology Co. Ltd.] to further confirm pathogen infiltration:

Primer-F: CCATCATCAAAGTTAAAGGCCAC.

Primer-R: GAGGAGGTTAGAATTTCATTTCGG.

The PCR reaction conditions were as follows: 3 min at 95 °C, followed by 40 cycles of 10 s denaturation at 95 °C, 30 s annealing at 60 °C, 30 s extension at 72 °C and 5 min final extension at 72 °C, using a thermal cycler [PX2^®^, Thermo™, USA]. The product was 514 bp in length. Finally, gel electrophoresis for amplified PCR products and the sequencing results of Sanger method were confirmed as positive.

## Discussion and conclusions

PAM is an acute, rapid and fatal disease of the central nervous system caused by *N. fowleri* infection, prevalent mainly in children and adolescents who swim in polluted ponds or swimming pools. Approximately 440 cases of this disease has been reported worldwide, most of which have occurred within the United States, Australia and Europe [5, 6], though very few PAM cases were reported in China [7]. The clinical manifestations of PAM are similar to those of bacterial meningitis, including severe headaches, high fever, projectile vomiting, seizures, neck stiffness, changes in the level of consciousness and meningeal irritation. Within 1–12 days after the onset of initial symptoms, the condition progresses rapidly and patients often die from increased intracranial pressure and brain herniation [8, 9].

This case occurred in a seaside city in southern China, where the patient went swimming three days prior to onset of symptoms, which is consistent with the incubation period reported in the literature [3]. In this case, the patient first experienced headaches, vomiting and high fever, followed by rapid seizures, coma and finally brain death. The changes in CSF were similar to those observed in bacterial meningitis. Unenhanced head MRI highlighted diffuse cerebral edema and cerebellar tonsil hernia, which were consistent with experiences previously reported by PAM patients [10]. The condition of the patient in this case progressed rapidly and multiple antibiotic treatments were ineffective. Both the CSF and blood high-throughput sequencing results reported *N. fowleri* infiltration, further confirmed through PCR and Sanger sequencing. We suggest clinicians should ideally investigate for less common potential pathogens, in patients with bacterial meningitis who are unresponsive to first-line antibiotic treatments.

In this particular case report, *N. fowleri* was also found to be present in blood, although its sequence copy number was lower for CSF. This finding suggests that *N. fowleri* could be transmitted outside the central nervous system, which was consistent with the study of two out of the five extra-CNS tissue from autopsies examined by the CDC in the United States from 2009 to 2012. In addition to the central nervous system, *N. fowleri* was also found in the lungs, kidneys, liver, spleen and other organs of four cases reported literature [11], we speculate that *N. fowleri* can enter the bloodstream through the damaged blood–brain barrier and progress to other tissues and organs through the bloodstream, though this requires validation.

Clinically, we emphasize the early identification of less-common pathogens for this form of rare and life-threatening brain infection, in order to achieve an early diagnosis and consequent treatment to improve patient prognosis. Furthermore, the employment of mNGS can be deemed to act as a vital diagnostic tool for rapid and accurate etiological detection of special/rare/unexpected/challenging to detect pathogens using traditional methods, such as PAM and other similar life-threatening infective conditions.

## Data Availability

All data generated or analyzed during this study are included in this published article. The data of mNGS and PCR are upload to NCBI (mNGS Accession numbers: SRR14251192, SRR14251191; Sanger sequences Accession numbers: MW965500, MW965501).

## Abbreviations

PAM: Primary amoebic meningoencephalitis
*N. fowleri*: *Naegleria fowleri*
mNGS: Meta-genomic next-generation sequencing
CSF: Cerebrospinal fluid
AmpB: Amphotericin B
CDC: Centers for Disease Control and Prevention
MRI: Magnetic resonance imaging
